# The impact of motility on the localization of *Lactobacillus agilis* in the murine gastrointestinal tract

**DOI:** 10.1186/s12866-018-1219-3

**Published:** 2018-07-11

**Authors:** Akinobu Kajikawa, Shunya Suzuki, Shizunobu Igimi

**Affiliations:** Department of Applied Biology and Chemistry, Tokyo University of Agriculture, 1-1-1 Sakuragaoka, Setagaya, Tokyo, 156-8502 Japan

**Keywords:** *Lactobacillus*, Motility, Flagella, Colonization

## Abstract

**Background:**

While the overall composition of the mammalian gut microbiota has been intensively studied, the characteristics and ecologies of individual gut species are incompletely understood. Lactobacilli are considered beneficial commensals in the gastrointestinal mucosa and are relatively well-studied except for the uncommon species which exhibit motility. In this study, we evaluate the importance of motility on gut colonization by comparing motile and non-motile strains of *Lactobacillus agilis* in mice models.

**Results:**

A flagellated but non-motile *L. agilis* strain was constructed by mutation of the *motB* gene. Colonization of the wild type and the mutant strain was assessed in both antibiotic-treated female Balb/c mice and gnotobiotic mice. The results suggest that the motile strain is better able to persist and/or localize in the gut mucosa. Chemotaxis assays indicated that the motile *L. agilis* strain is attracted by mucin, which is a major component of the intestinal mucus layer in animal guts.

**Conclusions:**

Motility and chemotactic ability likely confer advantages in gut colonization to *L. agilis*. These findings suggest that the motile lactobacilli have unique ecologies compared to non-motile commensals of the lactic acid bacteria.

**Electronic supplementary material:**

The online version of this article (10.1186/s12866-018-1219-3) contains supplementary material, which is available to authorized users.

## Background

Next generation sequencing technology has unveiled the diverse nature of the gut microbiota [1–3]. Albeit recent intensive studies reported key functions of these complex microbial communities [4–6], the ecology and roles of individual microbial species in the gastrointestinal (GI) tract have not been elucidated in detail. Lactic acid bacteria are culturable and beneficial microorganisms residing in animal guts, and thus their ecologies are relatively well-studied [7, 8]. Most of those lactic acid bacteria are non-motile, but a few members of the lactobacilli possess flagella and exhibit motility [9–12]. It is obvious that motility is not essential for gut colonization, which raises the question of why the energy-consuming machinery is maintained, while in most other members it has been lost during evolution. The most likely explanation is that motility provides certain advantages on survivability and persistence for these organisms in the gut mucosa. Hence, in this study we hypothesize that the motility of these lactobacilli strains contributes to colonization in the gastrointestinal tract.

*Lactobacillus ruminis* and *Lactobacillus agilis* are motile lactobacilli isolated from the GI tract of mammals [13–15]. Since established genetic tools are available [16], the latter one seems to be less difficult to use as a model microbe for analysis. In the current study, we have been able to construct a non-motile derivative strain from *L. agilis* BKN88, a highly motile strain [17]. This mutant is flagellated but lacks motility due to malfunction of a motor-switch protein. In two different murine models and in vitro assays, the colonization, localization, and chemotactic abilities of the motile and non-motile *L. agilis* strains were compared.

## Results

### Construction and validation of a *motB* (D23A) mutant of *L. agilis*

**Fig. 1:**
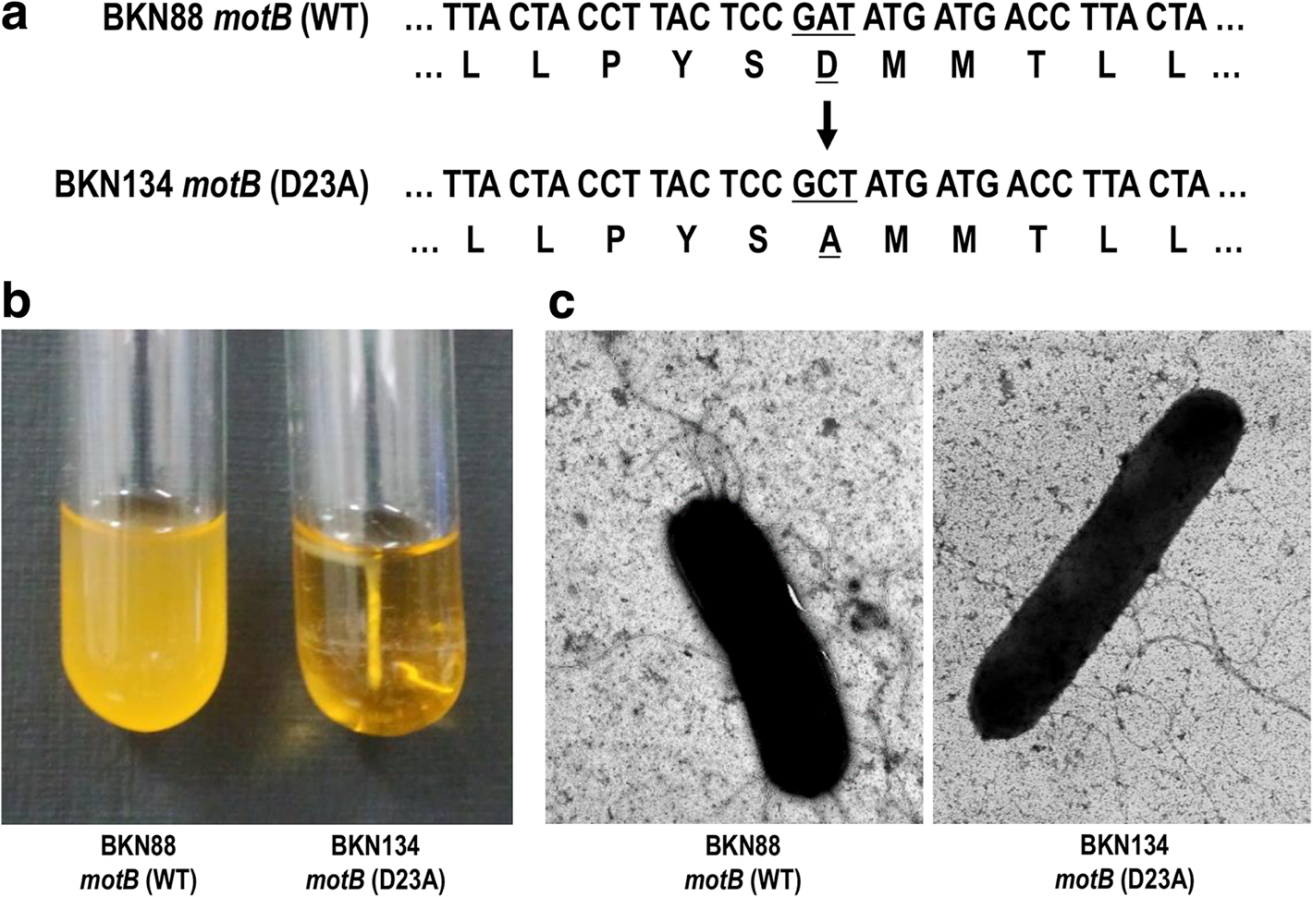
Flagellated but non-motile mutant of *L. agilis*. The nucleotide/amino acid sequences of the WT and mutated *motB* gene of *L. agilis* (**a**). Underlines represent the targeted codon/translation of the point mutation. Motility of *L. agilis* strains which have either WT or mutant *motB* gene (**b**). Overnight cultures of *L. agilis* BKN88 (WT) and BKN134 *motB* (D23A) in MRS-soft agar medium. Observation of flagella by electron microscopy (**c**). Flagellar filaments of BKN88 and BKN134 were visualized by negative staining


A consistently motile isolate of *L. agilis* JCM1048, originally isolated from a chicken, was obtained previously and designated BKN88 (Additional file 1). A non-motile derivative of *L. agilis* BKN88, was constructed through replacement of the wild type *motB* gene with a mutant *motB* (Fig. 1a). The resulting mutation, obtained by conversion of a single amino acid residue (23rd Asp to Ala), resulted in malfunction of MotB, the motor switch protein of the flagellar machinery. After sequence analysis confirmed the mutation of *motB* (D23A), the motility of the mutant strain, BKN134, was assessed in soft-agar culture. As shown in Fig. 1b, the mutant *L. agilis* strain exhibited no motility. This flagellar malfunction in BKN134 was also observed using optical microscopy (Additional file 2). The flagella were fully equipped in the mutant strain, and no structural difference was recognized between the wild type and the mutant strain (Fig. 1c).

**Additional file 1:** Microscopic analysis of motility of BKN88. (MP4 525 kb)



**Additional file 2:** Microscopic analysis of motility of BKN134. (MP4 583 kb)


### Antibiotic-assisted colonization of the *L. agilis* stains in mice

**Fig. 2:**
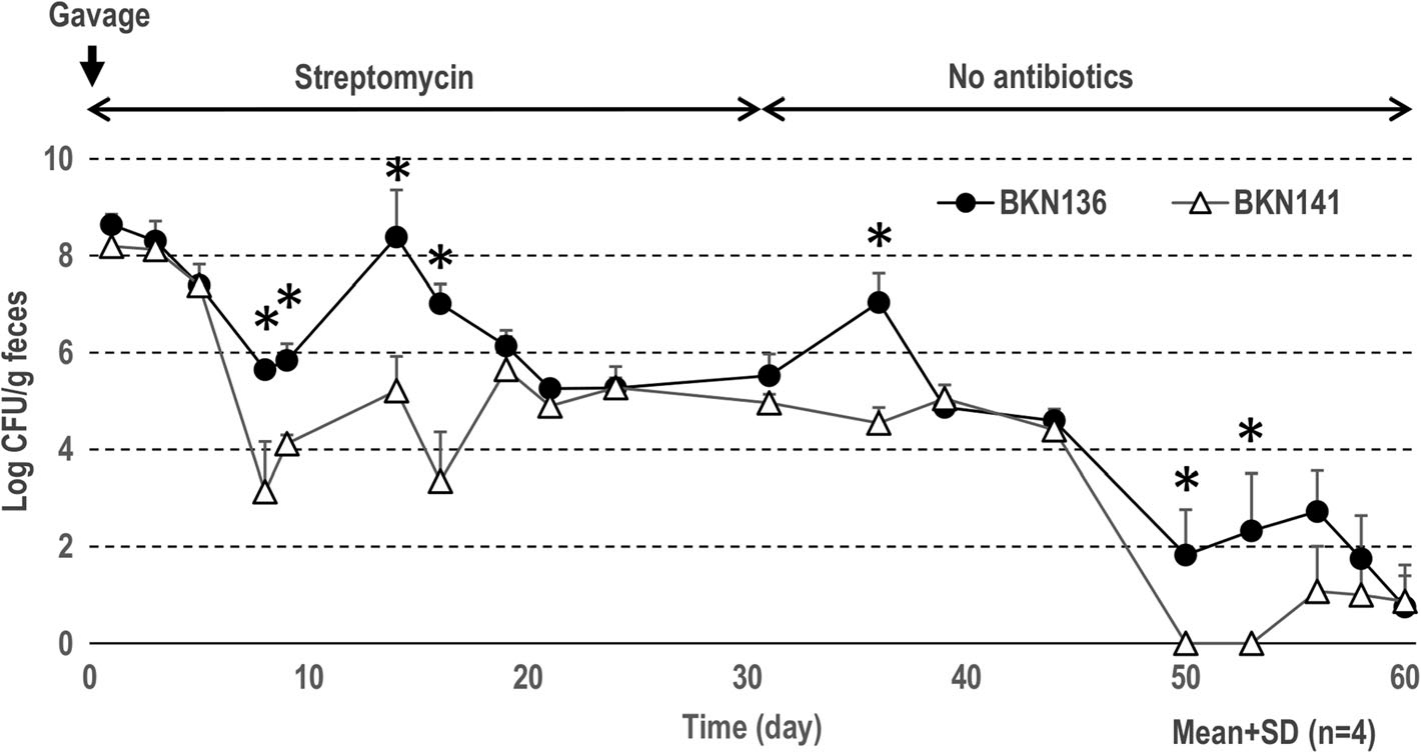
Antibiotic-assisted colonization of motile/non-motile *L. agilis* strains in Balb/c mice. CFU of streptomycin-resistant *L. agilis* strains in feces were tracked for 2 months. Mice were administered once with 1 × 10^9^ CFU of *L. agilis* by gavage. The animals received water supplemented with streptomycin during the first 30 days and no antibiotics for another 30 days. *P < 0.05

In a preliminary experiment, mice without antibiotic-treatment received *L. agilis* via the intragastric route; however, all *L. agilis* cells passed through the gastrointestinal tract within 2 days. For further experimentation, streptomycin resistant derivatives of BKN88 (BKN136) and BKN134 (BKN141) were isolated after growth on agar plates with 100 μg/ml of streptomycin. The streptomycin-resistant *L. agilis* strains were able to colonize for a reasonable duration in antibiotic-treated mice. Both strains were predominant in the first few days and then gradually decreased in number in mouse feces. As shown in Fig. 2, significantly higher numbers of motile *L. agilis* colonies were detected in comparison with the mutant for several of the data points. After removing antibiotics, the mice started shedding both *L. agilis* strains and eliminated all of the streptomycin resistant bacteria in a month. The motile or non-motile phenotype of the cells in the recovered colonies did not change throughout the experiment.

### Colonization of the *L. agilis* strains in Gnotobiotic mice

**Fig. 3:**
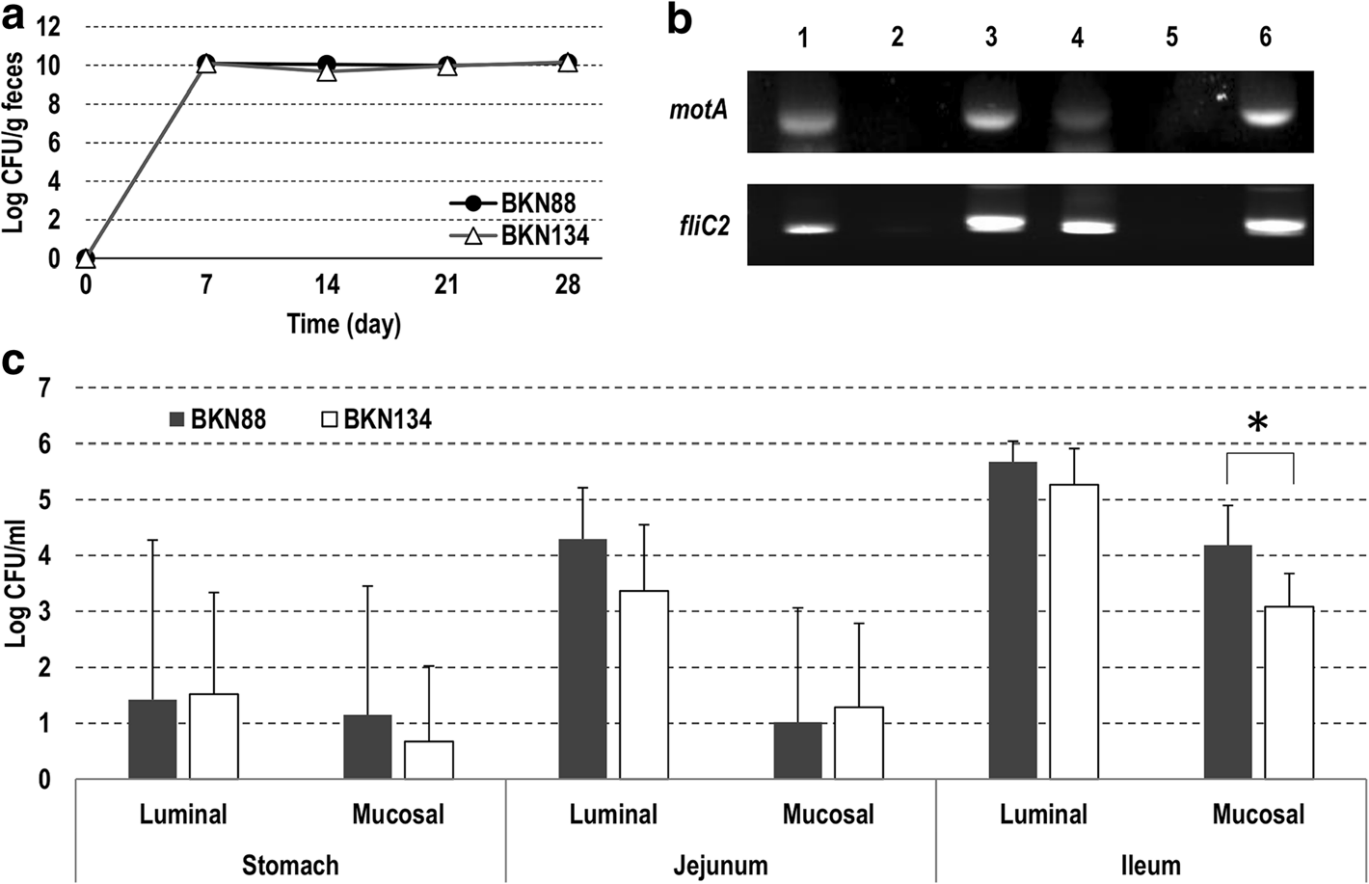
Colonization of motile/non-motile *L. agilis* strains in gnotobiotic mice. Gnotobiotic mice colonized by either BKN88 or BKN134 were kept in isolators for 4 weeks. CFU of the bacteria in feces were counted weekly (**a**). RT-PCR for detection of motility-associated gene-expression in vivo (**b**). BKN88 (Lane 1–3), BKN134 (Lane 4–6), lane 1 and 4: RT-PCR with total RNA isolated from cecal contents, lane 2 and 5: PCR with total RNA isolated from cecal contents, lane 3 and 6: PCR with chromosomal DNA isolated from bacterial culture grown in MRS-broth. CFU of luminal or mucosal bacteria in gastrointestinal tissues were counted (**c**). Mean value+SD (*n* = 4). *P < 0.05

Germ-free mice were administered the *L. agilis* strains and housed in isolators for a month. Fecal samples collected weekly stably maintained 10^10^ cfu/g of the lactobacilli throughout the experiment (Fig. 3a). No difference in numbers between motile and non-motile strains was found. After euthanasia, samples of the small intestines and ceca were collected. Total RNA was isolated from cecal contents, and the expression of the genes in the motility operon of *L. agilis* was confirmed by RT-PCR. As shown in Fig. 3b, both *motA* and *fliC2* were expressed in the murine gut. Numbers of lactobacilli in the local mucosa of small intestine samples were counted using roughly fractionated lavage fluids. Overall, the bacterial cells were more predominant in the upper gastrointestinal tract than the lower, and more predominant in the luminal fractions than mucus fractions (Fig. 3c). Significant differences were found only in the mucus fractions of the ilea. In this case, the occurrence of the motile strain was approximately 1-log higher than in the non-motile strain.

### Chemotaxis and penetration of the *L. agilis* strains in simulated mucus

**Fig. 4:**
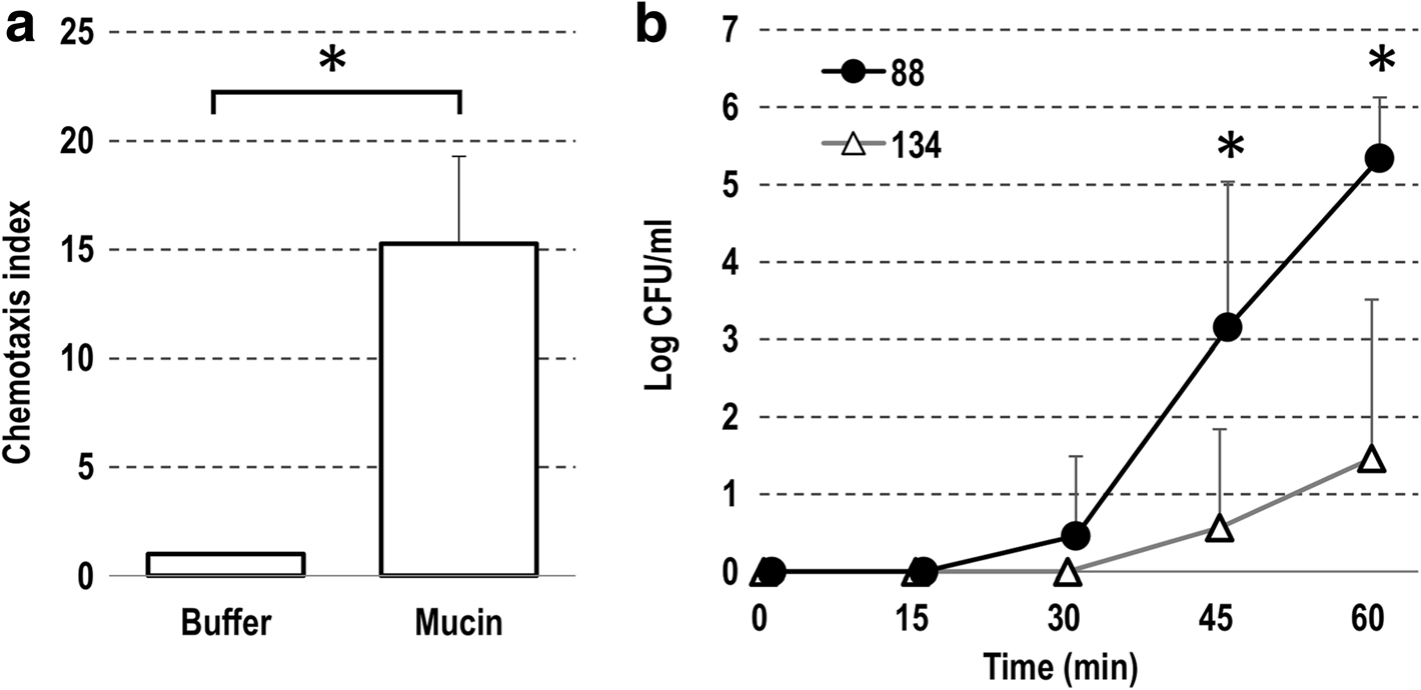
The motility of *L. agilis* toward mucin. Chemotaxis of *L. agilis* BKN88 attracted by mucin (**a**). Chemotaxis index designates relative cell numbers recovered from each capillary. The bar chart represents mean values (plus standard errors) of 5 independent assays. Translocation of the *L. agilis* strains through a simulated mucus layer (**b**). CFU of bacterial cells which passed through the simulated mucus layer was determined every 15 min. *P < 0.05

Mucin is a major component of the gastrointestinal mucus layer. A capillary assay was used to determine whether the motile *L. agilis* would exhibit chemotactic ability toward mucin. As shown in Fig. 4a, *L. agilis* BKN88 was clearly attracted by mucin. Penetration through the simulated mucus layer by the *Lactobacillus* strains was also assessed. While small numbers of the non-motile strain passively passed through the layer, the motile strain could penetrate the layer to a much greater extent (Fig. 4b).

## Discussion

The motility of flagellated enteropathogenic bacteria has been intensively investigated. These pathogens use the function to penetrate through mucus layers and invade host cells [18–20]. In return, the immune cells of the host recognize flagellar proteins via specific receptors such as TLR5 and NLRC4/IPAF to elicit innate immune responses [21–25]. Our previous work indicates that flagellins of *L. agilis* exhibit much lower immunological activity than those of major pathogenic bacteria [17]. This result implies that a host allows such bacteria to colonize as commensals. In the present study, we suggest that *L. agilis* takes advantage of its lower immunological activity in colonizing and/or localizing in the gut mucosa.

As described above, bacterial flagella possess at least two different functions, motility and immune-stimulating activity, Hence, a flagellated but non-motile strain was required to evaluate the exclusive impact of motility on colonization of the gastrointestinal mucosa of the host. A single amino acid mutation of the MotB protein confers a non-motile phenotype in *L. agilis* without loss of the flagella, as in a previous report in *L. monocytogenes* [26]. The currently constructed mutant seems to be an optimal strain to test our hypothesis.

Under antibiotic pressure, the streptomycin-resistant *L. agilis* strains could colonize the murine gut most likely because the bacteriocidal reagent substantially eliminated other competitive microbes. After discontinuing feeding of the antibiotic, *L. agilis* numbers reduced over time and eventually disappeared. In general, the motile *L. agilis* strain exhibited higher persistence in the gut than the non-motile mutant overtime. Meanwhile, similar amounts of bacterial cells were recovered regardless of motility in gnotobiotic mice, despite the fact that the flagella-associated genes were expressed in vivo. These dissimilar results among the two murine models seem conflicting, but might suggest that motility confers advantages on colonization only in case where the lactobacilli were surrounded by competitors. Albeit the total amount of lactobacilli in feces was similar in the gnotobiotic mice, the motile strain was detected in the mucosal/epithelial layer more frequently than the non-motile strain. Other experiments in vitro showed that only the motile *L. agilis* strain was attracted to mucin and had the ability to penetrate the mucus layer. Taken together, these results could support a hypothesis that the motile *L. agilis* cells actively localize in the middle of the mucus layer of the gut for robust colonization. Recent studies found that some gut microbes utilize mucin as a scaffold for cell-adhesion and/or as a carbon source [27–31]. Most lactobacilli in animal guts are also understood to utilize adhesins to attach to the local mucosa [32–35]. In contrast, this study suggests that *L. agilis* likely utilizes motility instead of or in addition to adhesion factors for its colonization.

We are aware of criticisms that the motile *L. agilis* strain recruited in this study is not a natural member of the gut microbiota of mice. Unfortunately, no motile lactobacilli have been isolated from mice or other rodents to the best of our knowledge. Thus, further studies need to be done in more appropriate animal models which include natural host-microbe combinations. Nevertheless, this study provides new and noteworthy insight into the ecology of motile lactic acid bacteria in the murine gut.

## Conclusions

We assessed the impact of motility on the colonization of *L. agilis* in the gastrointestinal mucosa in murine models. The results suggest that the bacteria could take advantage of motility to establish a niche which is likely distinct from other non-motile lactic acid bacteria. This study reveals an unexplored ecological feature of certain motile lactobacilli residing in animal guts.

## Methods

### Bacterial strains and growth conditions

*Lactobacillus agilis* BKN88 [17] and derivatives were grown statically (liquid culture) or anaerobically (plate culture) using AnaeroPouch-Anaero Anerobic Gas Generators (Mitsubishi Gas Chemical) in MRS broth/agar (Difco/BD) with or without 5 μg/ml of erythromycin or 100 μg/ml streptomycin at 37 °C. *E. coli* mc1061 was propagated aerobically in LB broth/agar with or without 200 μg/ml of erythromycin. All strains used in this study are described in Table 1. Motilities of *Lactobacillus* strains were determined by visual examination after inoculation into semi-solid MRS medium with 0.2% agar. Bacterial motility was also observed using an optical microscope (Keyence, Osaka, Japan).

**Table. 1:** Bacterial strains used in this study

Strain	Description	Reference
*E. coli*		
mc1061	Cloning host for pG^+^host5	Lucigen Co.
BKN-TB1	pG^+^host5::*motB* (D23A)-harboring strain, Em^r^	This study
*L. agilis*		
BKN88	Uniformly motile subculture of JCM 1048, Chicken isolate	[17]
BKN126	pG^+^host5::*motB* (D23A)-integrated intermediate	This study
BKN134	*motB* (D23A), Non-motile derivative of BKN88	This study
BKN136	Sm^r^ mutant derived from BKN88	This study
BKN141	Sm^r^ mutant derived from BKN134	This study

### Transmission electron microscopy (TEM)

Bacterial cells at exponential phase (OD_600_ = 0.8) were collected from liquid culture in MRS-broth. The bacterial cells and the flagellar filaments were negatively stained and visualized using a transmission electron microscope (JEM1200EX, JEOL Ltd., Tokyo, Japan) at 80 kV. This experiment was done by Hanaichi UltraStructure Research Institute (Aichi, Japan).

### Construction of a *motB* mutant of *L. agilis*

In *Listeria monocytogenes*, a single amino acid (23rd aspartate) replacement in MotB protein resulted in a non-motile phenotype without loss of the flagella structure [26]. *L. agilis* possesses an orthologous protein, and the specific amino acid residue is conserved. A DNA fragment containing mutant *motB* (D23A) and flanking region was generated by overlap PCR. Two separately amplified DNA fragments, an upstream fragment (Primer pair: DOKJ4, ATA TGG ATC CAG GAT TAT TAG CGC TAG AGG, and DOKJ7, AGG TCA TCA TAG CGG AGT AAG GTA GTA ACC) and a downstream fragment (Primer pair: DOKJ6, TAC TCC GCT ATG ATG ACC TTA CTA TTA TCC, and DOKJ5, ATA TGA ATT CAG CGG TAT CGT TAC TTG C), were assembled by subsequent PCR using DOKJ4 and DOKJ5. This PCR product was then digested with *Bam*HI and *Eco*RI followed by insertion into pG^+^host5 [36] using *E. coli* mc1061 as a cloning host. The constructed plasmid, pG^+^host5::motB (D23A), was introduced into *L. agilis* BKN88 by electroporation in accordance with a protocol reported by Stephenson et al. [16]. A *L. agilis* isolate with the integrated plasmid at the target locus, BKN126, was selected. The integrated pG^+^host5 with wild type *motB* gene was then excised, and a non-motile isolate, BKN134, was selected. The replacement of *motB* sequence was confirmed by sequencing.

### Colonization of *L. agilis* in antibiotic-treated mice

Mice were housed and cared for in accordance with the committee for the assessment of laboratory animal care standards and the guidelines of Tokyo University of Agriculture. To discriminate *L. agilis* from other gut microbes, naturally occurred streptomycin-resistant strains were isolated by plating *L. agilis* cultures onto MRS-agar containing 100 μg/ml of streptomycin. The antibiotic-resistant *L. agilis* were derived from either motile (BKN136) or non-motile (BKN141) strains. Female Balb/c mice were obtained from Crea Japan, Inc. Mice (four mice per group) were gavaged with 1 × 10^9^ cfu of either motile or non-motile *L. agilis* strains. After the gavage, the mice received drinking water supplemented with 100 μg/ml of streptomycin. Fecal samples were collected twice a week and streptomycin resistant colonies were enumerated. Motility of randomly selected colonies (10 colonies/mouse) recovered from fecal samples was tested periodically. After a month, streptomycin was removed from the drinking water. Before starting this experiment, no streptomycin-resistant bacteria was detected from the feces.

### Colonization of *L. agilis* in gnotobiotic mice

Care of gnotobiotic mice and collection of samples were operated by Sankyo Labo Service Co. (Tokyo, Japan). This experimental design was approved by the ethical committee of the company. Six weeks old female germ-free Balb/c mice were administered with either *L. agilis* BKN88 or BKN134. Feces were collected once a week and the cfu of each strain was determined. Motility of isolated colonies was tested as described above. After a month, mice were euthanized to collect specimens: stomachs, jejuna, ilea, and ceca. Luminal contents of these tissues except for ceca were then washed with PBS followed by treatment with dithiothreitol (DTT, 1 mM)/ EDTA (5 mM)-supplemented PBS to collect epithelial/mucosal lavage fluids. After serial dilution, those were spread onto MRS-agar and incubated anaerobically until colonies appeared.

### RNA-isolation from cecal contents and RT-PCR

Cecal contents were suspended in DNA/RNA Shield (Zymo Research) immediately after collecting the samples. Total RNA was purified with ZR Soil/Fecal RNA MicroPrep (Zymo Research) in accordance with the manufacturer’s instructions. To detect expression of motility genes in vivo, upstream and downstream loci of the motility operon of *L. agilis*, *motA* and *fliC2* were recruited as target genes. Pairs of specific primers, DOKJ505 (ATC GTC AAG GGT GCC AAC) and DOKJ506 (TTT GCT TGA TGG TCT TAG G) for *motA*, DOKJ51 (TTT CGG TAC AGG TGC A) and DOKJ52 (CTT TCT TGA TAG CAG C) for *fliC2*, were used respectively. Reverse transcription followed by PCR was performed with PrimeScript One Step RT-PCR Kit (Takara). In order to check DNA-contamination, PCR was also carried out with Takara Ex-taq. PCR-products were analyzed by 2% agarose-gel electrophoresis.

### Chemotaxis assay

Chemotaxis of *L. agilis* BKN88 to mucin was tested as described by Worku et al. with minor modifications [37]. Glass microcapillary tubes of 10 μl capacity were filled with 1% mucin from porcine stomach (SIGMA-Aldrich) in chemotaxis buffer (0.1 M potassium phosphate, 0.1 M glucose, 0.5 M EDTA, in pure water) and then sealed at the upper end with plastic film. The capillaries were inserted into to 1.5 ml microtubes with bacterial cells at mid-log phase suspended in the chemotaxis buffer at a concentration of 10^6^ cells/ml. After 1 h incubations, the outside of the capillary tubes was washed intensively with PBS followed by collection of the inner liquid. After serial dilution, the bacterial suspensions were spread onto MRS-agar plate for enumeration.

### Penetration of simulated mucus layers

Simulated mucus layers were prepared as reported previously [38]. Briefly, 0.1 ml of simulated mucus, 0.5% melting agarose with 12.5% mucin from porcine stomach (SIGMA-Aldrich), was transferred into cell culture inserts (8.0 μm pore) in 24-well plates (Corning). After gelling, 0.2 ml of MRS-broth was overlaid on the mucus layer and 1.0 ml of bacterial suspension at mid-log phase (1.0 × 10^8^ cells/ml) in MRS-broth was added to the well-plate before incubation at 37 °C. At designated time points, 20 μl of liquid-phase was removed from the insert followed by dilution and plating on MRS-plates for enumeration.

## Abbreviations

CFU: Colony forming units
DNA: Deoxyribonucleic acid
DTT: Dithiothreitol
EDTA: Ethylene diamine tetra acetic acid
Em^r^: Erythromycin resistance
GI: Gastrointestinal
LB: Luria-Bertani
MRS: Man, Rogosa and Sharpe
NLR: NOD-like receptor
OD: Optical density
PBS: Phosphate-buffered saline
PCR: Polymerase chain reaction
RNA: Ribonucleic acid
RT-PCR: Reverse Transcription-Polymerase chain reaction
Sm^r^: Streptomycin resistance
TEM: Transmission Electron Microscope
TLR5: Toll-like receptor 5
WT: Wild type

## References

[1] Qin J, Li R, Raes J, Arumugam M, Burgdorf KS, Manichanh C, Nielsen T, Pons N, Levenez F, Yamada T, Mende DR, Li J, Xu J, Li S, Li D, Cao J, Wang B, Liang H, Zheng H, Xie Y, Tap J, Lepage P, Bertalan M, Batto J-M, Hansen T, Le Paslier D, Linneberg A, Nielsen HB, Pelletier E, Renault P (2010). A human gut microbial gene catalogue established by metagenomic sequencing. Nature.

[2] Arumugam M, Raes J, Pelletier E, Le Paslier D, Yamada T, Mende DR, Fernandes GR, Tap J, Bruls T, Batto J-M, Bertalan M, Borruel N, Casellas F, Fernandez L, Gautier L, Hansen T, Hattori M, Hayashi T, Kleerebezem M, Kurokawa K, Leclerc M, Levenez F, Manichanh C, Nielsen HB, Nielsen T, Pons N, Poulain J, Qin J, Sicheritz-Ponten T, Tims S (2011). Enterotypes of the human gut microbiome. Nature.

[3] Lozupone CA, Stombaugh JI, Gordon JI, Jansson JK, Knight R (2012). Diversity, stability and resilience of the human gut microbiota. Nature.

[4] Turnbaugh PJ, Ley RE, M a M, Magrini V, Mardis ER, Gordon JI (2006). An obesity-associated gut microbiome with increased capacity for energy harvest. Nature.

[5] Hsiao EY, McBride SW, Hsien S, Sharon G, Hyde ER, McCue T, Codelli JA, Chow J, Reisman SE, Petrosino JF, Patterson PH, Mazmanian SK (2013). Microbiota modulate behavioral and physiological abnormalities associated with neurodevelopmental disorders. Cell.

[6] Clemente JC, Ursell LK, Parfrey LW, Knight R (2012). The impact of the gut microbiota on human health: an integrative view. Cell.

[7] Walter J (2008). Ecological role of lactobacilli in the gastrointestinal tract: implications for fundamental and biomedical research. Appl Environ Microbiol.

[8] Pessione E (2012). Lactic acid bacteria contribution to gut microbiota complexity: lights and shadows. Front Cell Infect Microbiol.

[9] Harriso AP, Hansen PA (1950). A motile *Lactobacillus* from the Cecal feces of turkeys. J Bacteriol.

[10] Ellsabeth Sharpe M, Latham MJ, Garvie EI (1973). Two new species of *Lactobacillus* isolated from the bovine rumen, *Lactobacillus* ruminis sp.nov. and *Lactobacillus* vitulinus sp.nov. J Gen Microbiol.

[11] Endo A, Okada S (2005). *Lactobacillus* satsumensis sp. nov., isolated from mashes of shochu, a traditional Japanese distilled spirit made from fermented rice and other starchy materials. Int J Syst Evol Microbiol.

[12] Irisawa T, Okada S (2009). *Lactobacillus* sucicola sp. nov., a motile lactic acid bacterium isolated from oak tree (Quercus sp.) sap. Int J Syst Evol Microbiol.

[13] O’ Donnell MM, Harris HMB, Lynch DB, Ross RP, O’Toole PW (2015). *Lactobacillus* ruminis strains cluster according to their mammalian gut source. BMC Microbiol.

[14] Yu X, Jaatinen A, Rintahaka J, Hynönen U, Lyytinen O, Kant R, Åvall-Jääskeläinen S, Von Ossowski I, Palva A (2015). Human gut-commensalic *lactobacillus* ruminis ATCC 25644 displays sortase-assembled surface piliation: phenotypic characterization of its fimbrial operon through in silico predictive analysis and recombinant expression in lactococcus lactis. PLoS One.

[15] Casey PG, Casey GD, Gardiner GE, Tangney M, Stanton C, Ross RP, Hill C, Fitzgerald GF (2004). Isolation and characterization of anti-salmonella lactic acid bacteria from the porcine gastrointestinal tract. Lett Appl Microbiol.

[16] Stephenson DP, Moore RJ, Allison GE (2011). Transformation of, and heterologous protein expression in, *Lactobacillus* agilis and *Lactobacillus* vaginalis isolates from the chicken gastrointestinal tract. Appl Environ Microbiol.

[17] Kajikawa A, Midorikawa E, Masuda K, Kondo K, Irisawa T, Igimi S, Okada S (2016). Characterization of flagellins isolated from a highly motile strain of *Lactobacillus* agilis. BMC Microbiol.

[18] Chaban B, Hughes HV, Beeby M (2015). The flagellum in bacterial pathogens: for motility and a whole lot more. Semin Cell Dev Biol.

[19] Feldman M, Bryan R, Rajan S, Scheffler L, Brunnert S, Tang H, Prince A, Scheffler LEE (1998). Role of flagella in pathogenesis of Pseudomonas aeruginosa pulmonary infection role of flagella in pathogenesis of Pseudomonas aeruginosa pulmonary infection. Infect Immun.

[20] Szymanski CM, King M, Haardt M, Armstrong GD (1995). Campylobacter jejuni motility and invasion of Caco-2 cells. Infect Immun.

[21] Hayashi F, Smith KD, Ozinsky A, Hawn TR, Yi EC, Goodlett DR, Eng JK, Akira S, Underhill DM, Aderem A (2001). The innate immune response to bacterial flagellin is mediated by toll-like receptor 5. Nature.

[22] Franchi L, Amer A, Body-Malapel M, Kanneganti T-D, Ozören N, Jagirdar R, Inohara N, Vandenabeele P, Bertin J, Coyle A, Grant EP, Núñez G (2006). Cytosolic flagellin requires Ipaf for activation of caspase-1 and interleukin 1beta in salmonella-infected macrophages. Nat Immunol.

[23] Miao EA, Alpuche-Aranda CM, Dors M, Clark AE, Bader MW, Miller SIAA (2006). Cytoplasmic flagellin activates caspase-1 and secretion of interleukin 1beta via Ipaf. Nat Immunol.

[24] Miao EA, Andersen-Nissen E, Warren SE, Aderem A (2007). TLR5 and Ipaf: dual sensors of bacterial flagellin in the innate immune system. Semin Immunopathol.

[25] Zhao Y, Yang J, Shi J, Gong YN, Lu Q, Xu H, Liu L, Shao F (2011). The NLRC4 inflammasome receptors for bacterial flagellin and type III secretion apparatus. Nature.

[26] O’Neil HS, Marquis H (2006). Listeria monocytogenes flagella are used for motility, not as adhesins, to increase host cell invasion. Infect Immun.

[27] Turroni F, Bottacini F, Foroni E, Mulder I, Kim J-H, Zomer A, Sanchez B, Bidossi A, Ferrarini A, Giubellini V, Delledonne M, Henrissat B, Coutinho P, Oggioni M, Fitzgerald GF, Mills D, Margolles A, Kelly D, van Sinderen D, Ventura M (2010). Genome analysis of Bifidobacterium bifidum PRL2010 reveals metabolic pathways for host-derived glycan foraging. Proc Natl Acad Sci.

[28] Van Den Abbeele P, Belzer C, Goossens M, Kleerebezem M, De Vos WM, Thas O, De Weirdt R, Kerckhof FM, Van De Wiele T (2013). Butyrate-producing Clostridium cluster XIVa species specifically colonize mucins in an *in vitro* gut model. ISME J.

[29] Derrien M, Vaughan EE, Plugge CM, de Vos WM (2004). Akkermansia municiphila gen. Nov., sp. nov., a human intestinal mucin-degrading bacterium. Int J Syst Evol Microbiol.

[30] Tailford LE, Crost EH, Kavanaugh D, Juge N (2015). Mucin glycan foraging in the human gut microbiome. Front Genet.

[31] Huang JY, Lee SM, Mazmanian SK (2011). The human commensal Bacteroides fragilis binds intestinal mucin. Anaerobe.

[32] Rojas M, Ascencio F, Conway PL (2002). Purification and characterization of a surface protein from *Lactobacillus* fermentum 104R that binds to porcine small intestinal mucus and gastric mucin purification and characterization of a surface protein from *Lactobacillus* fermentum 104R that binds to. Appl Environ Microbiol.

[33] Buck BL, Altermann E, Svingerud T, Klaenhammer TR (2005). Functional analysis of putative adhesion factors in *Lactobacillus* acidophilus NCFM Functional Analysis of Putative Adhesion Factors in *Lactobacillus* acidophilus NCFM. Appl Environ Microbiol.

[34] Kinoshita H, Uchida H, Kawai Y, Kawasaki T, Wakahara N, Matsuo H, Watanabe M, Kitazawa H, Ohnuma S, Miura K, Horii A, Saito T (2008). Cell surface *Lactobacillus* plantarum LA 318 glyceraldehyde-3-phosphate dehydrogenase (GAPDH) adheres to human colonic mucin. J Appl Microbiol.

[35] Granato D, Bergonzelli GE, Pridmore RD, Marvin L, Rouvet M, Corthe E (2004). Cell surface-associated elongation factor Tu mediates the attachment of *Lactobacillus* johnsonii NCC533 (La1) to human intestinal cells and mucins. Infect Immun.

[36] Biswas I, Gruss A, Ehrlich SD, Maguin E (1993). High-efficiency gene inactivation and replacement system for gram-positive bacteria. J Bacteriol.

[37] Worku ML, Karim QN, Spencer J, Sidebotham RL (2004). Chemotactic response of helicobacter pylori to human plasma and bile. J Med Microbiol.

[38] Lehker MW, Sweeney D (1999). Trichomonad invasion of the mucous layer requires adhesins, mucinases, and motility. Sex Transm Infect.

